# Coping with the COVID-19 crisis: an overview of service adaptation and challenges encountered by a rural Psychiatry of Later Life (POLL) team

**DOI:** 10.1017/ipm.2020.86

**Published:** 2020-07-02

**Authors:** M. Usman, S. Fahy

**Affiliations:** St Brendan’s CNU, Creagh, Ballinasloe, Co. Galway, Republic of Ireland

**Keywords:** COVID-19, pandemic, Psychiatry of Later Life, rural, telepsychiatry

## Abstract

The COVID-19 pandemic has posed many challenges in maintaining standards of care and treatment for patients while managing the increased anxieties of patients, carers and the public in general. This paper highlights several clinical, administrative, medicolegal and IT implications of COVID-19 on the delivery of mental healthcare to an elderly vulnerable patient cohort due to recommended social distancing measures. Our Psychiatry of Later Life team has adapted to this by restricting face-to-face consultation, while continuing to provide telephone support. We have modified our documentation standard and have improved some aspects of our team working by facilitating flexible working arrangement and relevant training for staff as well as by embracing new technology. Notwithstanding the challenges therefore, this exceptional time has also opened avenues for new and innovative opportunities that can be further explored even when the current crisis eventually passes.

## Introduction

With the onset of the COVID-19 pandemic, there have been unprecedented changes in world dynamics in a matter of months. Consequently, the delivery of healthcare across the globe has been drastically affected. In order to keep up with the rapidly evolving situation, and the plethora of information that has been issued since the onset of the pandemic, it has been necessary to look at novel ways of adapting to the new circumstances in mental healthcare. It is also important to bear in mind that while we aim for perfection in standards of care and documentation, this may not be achievable in these challenging times. We believe that a consistency of approach is necessary in relation to patient care, functioning of the multidisciplinary team (MDT) and administrative standards while addressing ‘contingency standards of care’ (Ethical Considerations Relating to Critical care in the Context of COVID-19 from Department of Health, 2020).

## Clinical perspective

The Community Mental Health Teams have had to quickly adapt to the challenges posed both by the pandemic and by the public health measures to control it (Lyne *et al.* 2020). As the Psychiatry of Later Life (POLL) team caters for the mental health needs for the elderly population, our clients are some of those who are most at risk of the associated complications from the COVID-19 virus because of their age, comorbid medical conditions and/or being in a residential care facility (RCF) (HSE.ie/ Coronavirus 2020; CDC.gov/ Coronavirus Disease 2020). Consequently, our team decided very early on in the course of this pandemic to restrict our face-to-face (F2F) contact with our elderly vulnerable population. This was managed by providing triage of all referrals and where appropriate, telephone assessments were offered in lieu of F2F assessments. We believe that telepsychiatry has potential to provide quality care while minimizing the risk of infection to staff and patients (Whaibeh *et al.* 2020).

Not surprisingly, all patients contacted by the team expressed concerns about the risk of contracting COVID-19. Currently, however, the virus has not yet affected large cohorts of people in our catchment area. As of 8th June, 2020, the total number of confirmed cases of COVID-19 in the Republic of Ireland was 25 207, while 481 cases were confirmed in Co. Galway, which was in the lower half of statistical data of confirmed cases for all counties (HSPC.ie/ Covid-19 Cases in Ireland 2020).

Encouragingly, our patients have adapted well to receiving support by phone. Most have expressed gratitude for the contact and placed a value on regular phone contact at a pre-agreed time and day each week. Additionally, we have observed that older adults generally prefer phone call over video contact when given a possible choice of a telephone call or a WhatsApp video call. We now have access to HSE-supported video call facilities, that is, Attend Anywhere and have used this successfully with our patients.

The greatest challenge posed for our client group is for those who live alone or in isolated areas with no family close by. Most of our clients live rurally. All day centers for older people are currently closed so this form of social support is not available. Logistically, most of our patients are retired and may have family or neighbors for support. Their daily habits have been restricted and cocooning has also been a challenge for those who enjoyed their independence and outdoor activities prior to the lockdown (Plagg *et al.* 2020). On the other hand, patients who were contented to be at home before the restrictions have coped well with cocooning.

MDT members have given feedback that the quality of therapeutic rapport is not as good in instances when initial and subsequent assessments are performed by phone as opposed to having already established a F2F connection. In situations where a team member knows a patient well and has established F2F rapport with them, the lines of communication are easier to maintain even on remote consultation. For the same reason, our patients have responded very well to maintenance cognitive stimulation therapy intervention provided by phone like relaxation, arts and crafts, reminiscence therapy, puzzles and crosswords, suggested television programmes, newspaper and radio. However, the challenge is to gauge and interpret nonverbal responses on a phone call, as we cannot observe body language. Clinicians have been concerned with interpretations of nuances and emotions in remote consultations which they believe can in turn have an impact on rapport building (Cowan *et al.* 2019). Many studies, both large and small, found no significant differences in the therapeutic alliance when the care is delivered F2F with care delivered through videoconferencing (Cook and Doyle, 2002; O’Reilly *et al.* 2007; Bouchard *et al.* 2010; Jenkins-Guarnieri *et al.* 2015). However, when compared between the providers and the receivers, there is evidence that clinicians are more concerned about the potential of impaired therapeutic rapport with telepsychiatry than the patients (Hubley *et al.* 2016; Lopez *et al.* 2019). There are studies that show a superiority of videoconferencing over telephone consultation in different medical specialties (Pinnock *et al.* 2003; Handschu *et al.* 2008; Donaghy *et al.* 2019) and many studies show similar outcome for telephone consultation when compared with F2F consultations in psychiatric practice (Rohde *et al.* 1997; Aziz and Kenford, 2004; Bains *et al.* 2010; Hajebi *et al.* 2012). A particular issue in elderly populations is the lack of familiarity with the technology. A study by Berner (2014) showed that increased age, lower education or cognitive status, living alone or in rural areas and frailty are indicators of less internet use. They suggested that touch screen devices are therefore more user friendly. We have also carried out a separate project of using Kindles with our patients in an RCF and we have found that patients engage well with the activities or their old photos on the Kindle device with assistance from staff. Another study found that older adults are more likely to use the technology if they perceive it’s beneficial, easy-to-use, affordable and compatible with their daily schedules (Lee, 2014).

The interventions by Allied Health Professionals have also been restricted in that their potential input and services provided are limited during this pandemic, for example, deep psychological work like exploring past trauma or challenging of defenses that are emotionally triggering for patients is not appropriate during current crises due to limitations in providing a safe therapeutic space or use of nonverbal therapeutic techniques. The therapeutic focus has therefore changed to ‘holding’ the client and prioritizing maintaining/improving emotional stability and promoting coping skills. Another role taken on by the team is provision of updated accurate information if required about social distancing, cocooning and self-isolation as well as maintaining connection with the ‘outside world’ in the case of those who are socially or geographically isolated (Van Orden *et al.* 2020).

Another impact of COVID-19 pandemic is on the pattern of the number of referrals to our service. Our referral rate is generally 50–60 referrals per month (55 in January 2020). However, immediately post the onset of COVID-19, we had a sharp drop in referral rate (19 in April 2020) but we now see referral numbers rising to and surpassing pre-COVID level. This finding is in line with a survey conducted by the College of Psychiatrists of Ireland entitled ‘Covid-19 Impact on Secondary Mental Healthcare Services in Ireland’ (June 2020) and would be predicted in stage 4 of pandemic epidemiology (hcldr.wordpress.com/ The Pandemic’s 4^th^ Wave 2020).

## Staff and MDT issues

A need for clear communication and up-to-date documentation cannot be overemphasized in this exceptional period especially when some team members are working from home and in a flexible manner due to social distancing or childcare responsibilities. As a team, we have drawn up a local protocol to triage all incoming referrals, to ensure role clarity for all MDT members in processing and managing referrals and to ensure standard of assessments and documentation are maintained. Traditionally, a core assessment was performed by a team doctor with community mental health nurse. However, the consultant arranged an in-service training on completion of Core Assessments for all MDT members if some team members developed COVID-19 or needed to self-isolate. At this point, most of the team members have performed initial assessments on their own followed up by a phone call from the doctor at a later time. All assessments continue to be discussed in full at the weekly MDT meeting with completion of an MDT care plan. These MDT meetings are carried out by Zoom conferencing to allow people to work from home and to encourage social distancing.

We devised a short GP letter template for core assessment and medical reviews to be completed following the phone consultation in case clerical input is not available. In cases where the patient is contacted by phone and charts are not available to the relevant team member, it was agreed that their entries would be made when they are present on site (as retrospective notes). Alternatively, some team members come in to complete their documentation at a time when less people are on site (e.g. evenings or weekends).

## Technology issues

We have used Zoom software for our team meetings. There have been concerns raised about the security around Zoom, Doxy.me and other technology platforms but following risk assessment it was decided to proceed with this until a more secure option became available nationally. We have trialed Blue Eye software for patient video calls, but the success of this has been intermittent and unreliable in our experience to date. ‘Attend Anywhere’ is the latest platform recommended and supported by the HSE and has been used successfully for video consultation with patients. Issues around ease of use have arisen when the patients have sensory deficits (hearing/visual), or difficulty in understanding the instructions to connect online but this problem is resolved if family members or carers are available to assist. Some of the challenges for older adults in adoption of technology are appreciation of its value, user friendliness, cost, confidence or compatibility with their daily lives. There should therefore be an emphasis on design and development of technology devices that meet the specific needs of older people. Their experience can be improved with tutorials and continued support that makes the technology less intimidating for them (Lee, 2014).

## Medicolegal issues

The Medical Protection Society (MPS) held webinars which addressed medicolegal issues when providing remote consultation during the pandemic. Some issues highlighted were the need to ensure patients’ identity, obtain verbal consent, maintain confidentiality, explain non-F2F consultation and check their ability to understand and retain the proposed management plan (Webinar recording: Remote Consulting during the COVID-19 outbreak, 2020). We devised a checklist of the MPS recommendations for use with all initial assessments. MDT members were also advised to be mindful of these issues in all medical/nursing reviews.

## Staff training

A very important aspect of this unprecedented situation is the volume of information and the fast pace of change in the management of COVID-19. To keep pace with the ever-changing situation, staff access to information, training and support is pivotal. Our team accessed training specific to COVID-19 signs and symptoms, personal protective equipment and hand hygiene. In addition, we also focused on adapted clinical skills training which involved training on Core Assessment for all MDT members, Irish National Early Warning Score (INEWS) training, Psychological First Aid for team members and the use of DBT skills in the current scenario. We have implemented INEWS assessments and escalation protocol flowchart for monitoring our patients in an elderly RCF to identify changes in vital function as early as possible.

## Challenges faced and lessons learned

The primary challenges we have faced as a team include the deluge of information, change-of-pace of criteria and management of the impact of the virus, and the ongoing provision of quality mental healthcare for our patients while minimizing the risk of infection to patients and staff. Furthermore, there is a need to support all team members and maintain efficient teamwork and social distancing.

Ironically, there have been some positives resulting from the COVID-19 pandemic. Some of the positive effects have been that we have learned new ways of working within our team and have seen evidence of improved staff collegiality. Team members support each other by offering to review patients if the involved professional is not available or overburdened. POLL service delivery previously involved most of the home assessments in-line with the HSE National Service Plan (2020) that encourages delivery of services for elderly citizens in their own homes according to their specific needs and preferences. Since the onset of the pandemic, the number of home assessments has reduced significantly. Consequently, the time spent on driving is greatly reduced.

Another positive outcome of the pandemic is the use of videoconferencing facilities for team meetings and training. We have facilitated flexible working hours, which has allowed team members to navigate the real challenges of sharing childcare with working partners. We have started using a team WhatsApp group for team notifications as well as for team support.

We plan to maintain some of these adapted ways of working post-pandemic, such as the option to use telepsychiatry for obtaining routine information with early F2F assessments subsequently to assess mental state or cognitive status. We intend to research the acceptability and ease of use of telepsychiatry in our cohort of patients over the coming weeks. We also plan to continue using videoconferencing facilities for some meetings and training events (to lessen unnecessary travel and time wastage). We plan to survey GP colleagues’ opinion on our revised shortened version of post-assessment letter to referrer and may adopt this shortened version ongoing, which places less burden on clerical staff. We have also found that the revised documentation is sent to referring agents in a timelier manner.

Despite the challenges posed by the COVID-19, we have endeavored to maintain optimum standards of care and are providing extra support to our patient population during this time, we have adapted our ways of team working and have modified our documentation standard to reflect the changes in our practice, we have enhanced the efficiency of some aspects of our team working and we have embraced innovation and technology. We have done all the above in anticipation of the longer-term effects of this pandemic and hope that our efforts will sustain the delivery of acute assessments and continuity of care for our vulnerable patients during this exceptional time and beyond.
